# Editorial: Insights in veterinary epidemiology and economics: 2023

**DOI:** 10.3389/fvets.2025.1564068

**Published:** 2025-02-13

**Authors:** Ioannis Magouras, Salome Dürr, Beatriz Martínez-López, Victoria J. Brookes

**Affiliations:** ^1^Veterinary Public Health Institute, Vetsuisse-Faculty, University of Bern, Bern, Switzerland; ^2^Department of Infectious Diseases and Public Health, Jockey Club College of Veterinary Medicine, City University of Hong Kong, Kowloon, Hong Kong SAR, China; ^3^Department of Medicine and Epidemiology, Center for Animal Disease Modeling and Surveillance (CADMS), School of Veterinary Medicine, University of California, Davis, Davis, CA, United States; ^4^Faculty of Science, Sydney School of Veterinary Science, The University of Sydney, Camperdown, NSW, Australia; ^5^Sydney Infectious Diseases Institute, The University of Sydney, Camperdown, NSW, Australia

**Keywords:** veterinary epidemiology and economics, data-driven approaches, One Health, innovation, transdisciplinarity, editorial

The field of veterinary epidemiology and economics continues to evolve, driven by a growing need to protect animal health, support sustainable food systems, and address emerging public health challenges in the face of climate change and global travel and trade (1, 2). This Research Topic brings together a collection of studies that highlight both recent advancements and ongoing challenges within the discipline. The studies demonstrate the importance of data-driven approaches, transdisciplinary collaboration particularly in a One Health context, and both refinement of existing tools and the development of innovative methodologies to address global animal health concerns.

Water quality remains an essential factor influencing livestock health and productivity. By conducting an integrated approach using aerobic mesophilic counts, pathogen isolation, and antimicrobial resistance testing to investigate the microbial quality of poultry drinking water on farms in Austria, Mustedanagic et al. revealed persistent contamination despite various water line treatments. Their findings underscore the complexity of eliminating opportunistic pathogens, such as *Pseudomonas* spp., and highlight the risks posed to both poultry and farm personnel. This research serves as a reminder of the critical need for effective water management strategies to mitigate antimicrobial resistance (AMR) and ensure poultry welfare and demonstrates how existing methods can be combined to provide additional insights to ongoing challenges.

The global challenge of AMR in both human and veterinary medicine was also a focus of a study by Marco-Fuertes et al. of non-traditional small companion mammals in Spain, which highlighted the role these animals may play as reservoirs for AMR *Staphylococci*. The findings, which show alarming levels of multidrug resistance, underscore the importance of a One Health approach to address the interconnected health of humans, animals, and the environment. Vigilant monitoring and interdisciplinary collaboration are essential to curtail the spread of resistant pathogens.

Planned investments in animal health are foundational to sustainable agricultural practices. Schrobback et al. explored on-farm investments into dairy cow health across 15 countries, offering insights into the allocation of resources for veterinary care and medicine. The study demonstrated that while direct health expenditures represent a modest portion of total production costs, their impact on overall productivity and animal welfare is significant. This research demonstrates how economic analyses are critical in directing benefits in animal health and provides valuable benchmarks for policymakers and farm managers aiming to optimize health investments in dairy production systems worldwide. In addition to ongoing health management, planning responses to infectious disease incursions and the fight against transboundary diseases such as foot-and-mouth disease (FMD) remain a priority in veterinary epidemiology. The theme of evidence-based decision making is continued in the Research Topic in a study by Cardenas et al., in which FMD spread was simulated in Brazil to evaluate control measures. This study demonstrated the use of a multi-host stochastic multilevel model and the value of real-world data to determine the effectiveness of rapid response strategies, including vaccination and depopulation. The findings provide critical insights for policymakers on managing FMD outbreaks and highlight the importance of preparedness and scalable interventions.

Expanding health planning to wildlife populations is another innovation highlighted in this Research Topic. In a perspective about utilizing livestock health planning models for wildlife management, Patterson identifies how structured health planning approaches that are commonly used in livestock management could be adapted to structured health plans for conservation and ecosystem health. While challenges remain in adapting such frameworks to wild populations, the perspective presents a compelling case for applying evidence-based planning to wildlife health initiatives to inform decision making around resource allocation and intervention implementation, to enhance wildlife health management, conservation and public health benefits through reduced opportunity for pathogen transmission.

Causal inference is a cornerstone of epidemiology, and its importance is highlighted through contributions that address its principles and the critical process of variable selection in veterinary epidemiology (Ruple et al.; Sargeant et al.). Sargeant et al. underscore the necessity of embracing causal inference by advocating for the articulation of clear hypotheses and the meticulous selection of confounding variables to ensure validity and reproducibility. Ruple et al. call for rigorous methodologies in exposure variable selection and validation to minimize measurement errors; when proxy variables are necessary, they should be thoughtfully chosen and transparently reported so evidence informing health policies and interventions is reliable and contributes to improved population health outcomes. Together, these studies highlight the need for adoption of more rigorous frameworks for observational research that would align veterinary epidemiological practices with the methodological rigor observed in other branches epidemiology.

Lastly, effective communication between researchers and stakeholders is crucial for translating scientific findings into actionable policies. Renter et al. advocate for a stakeholder-driven approach to promote the alignment of study outcomes with the practical needs of end-users and thus enhance the utility and impact of scientific findings. By using a case example in which interventions for bovine respiratory disease were evaluated, they highlight the need to considering multiple outcomes—such as antimicrobial use, animal welfare, and economic factors—to meet stakeholder decision-making requirements. Whether the beneficiaries are livestock owners, pet guardians, or public health officials, research outcomes must be relevant and reliable to inform decision-making processes effectively.

Collectively, these contributions underscore the dynamic and multifaceted nature of veterinary epidemiology and economics. They address critical contemporary challenges including antimicrobial resistance, the spread of transboundary diseases, methodological rigor in research, and effective stakeholder engagement. By offering new insights and practical solutions, this Research Topic advances the field and contributes to the broader objective of safeguarding animal and public health in an increasingly interconnected global landscape. Looking ahead, it is evident that collaboration, innovation, and a commitment to scientific rigor will be indispensable in tackling the complex challenges facing veterinary epidemiology and economics.
